# The Rac Activator DOCK2 Mediates Plasma Cell Differentiation and IgG Antibody Production

**DOI:** 10.3389/fimmu.2018.00243

**Published:** 2018-02-16

**Authors:** Miho Ushijima, Takehito Uruno, Akihiko Nishikimi, Fumiyuki Sanematsu, Yasuhisa Kamikaseda, Kazufumi Kunimura, Daiji Sakata, Takaharu Okada, Yoshinori Fukui

**Affiliations:** ^1^Division of Immunogenetics, Medical Institute of Bioregulation, Kyushu University, Fukuoka, Japan; ^2^Research Center for Advanced Immunology, Kyushu University, Fukuoka, Japan; ^3^Department of Biosciences, School of Science, Kitasato University, Sagamihara, Japan; ^4^Department of Pharmacology, Faculty of Medicine, University of Miyazaki, Miyazaki, Japan; ^5^Laboratory for Tissue Dynamics, RIKEN Center for Integrative Medical Sciences, Yokohama, Japan

**Keywords:** Rac activation, DOCK2, B cell receptor, immunological synapse, plasma cell, antibody production

## Abstract

A hallmark of humoral immune responses is the production of antibodies. This process involves a complex cascade of molecular and cellular interactions, including recognition of specific antigen by the B cell receptor (BCR), which triggers activation of B cells and differentiation into plasma cells (PCs). Although activation of the small GTPase Rac has been implicated in BCR-mediated antigen recognition, its precise role in humoral immunity and the upstream regulator remain elusive. DOCK2 is a Rac-specific guanine nucleotide exchange factor predominantly expressed in hematopoietic cells. We found that BCR-mediated Rac activation was almost completely lost in DOCK2-deficient B cells, resulting in defects in B cell spreading over the target cell-membrane and sustained growth of BCR microclusters at the interface. When wild-type B cells were stimulated *in vitro* with anti-IgM F(ab′)_2_ antibody in the presence of IL-4 and IL-5, they differentiated efficiently into PCs. However, BCR-mediated PC differentiation was severely impaired in the case of DOCK2-deficient B cells. Similar results were obtained *in vivo* when DOCK2-deficient B cells expressing a defined BCR specificity were adoptively transferred into mice and challenged with the cognate antigen. In addition, by generating the conditional knockout mice, we found that DOCK2 expression in B-cell lineage is required to mount antigen-specific IgG antibody. These results highlight important role of the DOCK2–Rac axis in PC differentiation and IgG antibody responses.

## Introduction

B cells play an important role in protective immunity through production of antibodies that bind to and eliminate foreign antigens. During development in the bone marrow (BM), precursor B cells undergo rearrangements of the gene encoding the B cell receptor (BCR) and differentiate into immature B cells, which migrate to the spleen to complete their development *via* T1 and T2 transitional stages (1, 2). Mature follicular B cells then enter secondary lymphoid tissues such as the lymph nodes (LNs) in search for cognate antigens. Specific recognition of antigen by the BCR triggers intracellular signaling cascades, leading to activation of mature B cells and differentiation into plasma cells (PCs) (3, 4). During T cell-dependent (TD) humoral immune responses, PCs are initially produced in transient extrafollicular proliferative foci, but are subsequently derived from B cells participating in the follicular germinal center (GC) reactions (5–7). Accumulating evidence indicates that low-affinity antigens fail to induce PC differentiation (8–10). However, its underlying mechanism and cellular response are poorly understood.

Although soluble antigens can activate B cells, membrane-bound antigens are more effective in promoting B cell activation and are likely to constitute the dominant form of antigens responsible for B cell stimulation *in vivo* (11). When a mature B cell recognizes antigens tethered on the surface of a target cell such as the follicular dendritic cell (FDC), a microcluster of BCR and its cognate antigen forms and grows at the site of the contact (4), which is surrounded by adhesion molecules, leukocyte function-associated antigen-1 (LFA-1), and intercellular adhesion molecule-1 (ICAM-1) on the surface of B cells and FDCs, respectively. This structure is known as immunological synapse (IS), and its formation involves membrane polarization and cytoskeletal reorganization (4). Previous studies have indicated that the affinity of the BCR for antigen affects the extent of antigen accumulation at the contact site (12, 13). Additionally, it is well established that intracellular signaling molecules also polarize to the IS, following a precise relative topology (4). Therefore, IS formation may be an important factor that determines the fate of antigen-specific B cells during humoral immune responses.

Rac is a member of Rho family GTPases that function as molecular switches by cycling between GDP-bound inactive and GTP-bound active states (14, 15). Rac exists in the cytosol in the GDP-bound form and is recruited to membranes, where its GDP is exchanged for GTP by the action of one or more guanine nucleotide exchange factors (GEFs) (14, 15). Once activated, Rac binds to multiple effector molecules and regulates various cellular functions including remodeling of the actin cytoskeleton. Rac is composed of three isoforms, Rac1, Rac2, and Rac3. Rac1 is ubiquitously expressed and Rac3 is highly expressed in the brain, whereas Rac2 expression is restricted largely to hematopoietic cells (15). So far, the role of Rac in B cells has been extensively analyzed using conventional Rac2 knockout (KO; *Rac2^–/–^*) mice and/or conditional KO mice lacking Rac1 expression in B cell lineage (16–18). These results have shown that Rac2 is more important than Rac1 in B cell development, B cell adhesion, and IS formation. However, the effect of loss of Rac activation on antibody production remains unknown, because genetic deletion of both Rac1 and Rac2 in B cell lineage leads to virtually complete absence of mature B cells (17).

DOCK2 is a member of the CDM family of proteins (*Caenorhabditis elegans* CED-5, mammals DOCK180, and *Drosophila melanogaster* Myoblast City) and is predominantly expressed in hematopoietic cells (19, 20). Although DOCK2 does not contain the pleckstrin homology (PH) and Dbl homology (DH) domains typically found in GEFs, DOCK2 can bind to phosphatidylinositol 3,4,5-triphosphate (PIP_3_) through its DOCK homology region (DHR)-1 domain and mediates the GTP–GDP exchange reaction for Rac by means of its DHR-2 domain (21–25). DOCK2 plays key roles in migration and activation of T cells, and its deficiency severely impairs humoral immune responses to TD antigens in mice and humans (26–29). However, the B cell-intrinsic role of DOCK2 in antibody production remains unknown. In this study, we found that BCR-mediated Rac activation and IS formation critically depend on DOCK2. By analyzing three different models, we demonstrate here that DOCK2 expression in B-lineage cells is required for PC differentiation and antigen-specific IgG production.

## Materials and Methods

### Mice

*Dock2^−/−^* mice on C57BL/6 background (CD45.2^+^) have been described previously (26–28). HyHEL10 mice were generated by crossing VDJ9 HyHEL10 heavy-chain knock-in mice with Vκ5 HyHEL10 light chain transgenic mice (30) and bred to congenic C57BL/6 mice carrying CD45.1^+^ allele. For adoptive transfer experiments, *Dock2*^+/^*^−^* or *Dock2^−/−^* HyHEL10 mice carrying CD45.1^+^ allele were generated. For development of DOCK2 conditional KO mice, ES cell harboring loxP-flanked exon 3 of *Dock2* allele (EUCOMM consortium) were microinjected into C57BL/6 blastocysts, and the male chimeras were crossed with C57BL/6 mice to obtain *Dock2^lox/lox^* mice (for details, see Figure S1 in Supplementary Material). *Dock2^lox/lox^* mice were crossed with CD19-Cre mice (CD19-Cre^+/^*^−^*), in which a *Cre* recombinase gene is inserted heterozygously into the first exon of the *CD19* (31).

### Cell Preparation and *In Vitro* Functional Assays

B cells were purified from the peripheral LN cells with B cell Isolation kit (Miltenyi Biotec) and cultured in RPMI 1640 medium (Wako) containing 10% heat-inactivated fetal calf serum (Nichirei Biosciences), 50 µM 2-mercaptoethanol (Nacalai tesque), 2 mM l-glutamine (Life Technologies), 100 U/ml penicillin (Life Technologies), 100 µg/ml streptomycin (Life Technologies), 1 mM sodium pyruvate (Life Technologies), and MEM non-essential amino acids (Life Technologies) (designated complete RPMI medium). For proliferation assay, LN B cells (5 × 10^4^/well) were stimulated in complete RPMI medium with the specified concentrations of anti-IgM F(ab′)_2_ antibody (Jackson ImmunoResearch Laboratories), anti-CD40 antibody (BD Biosciences), or lipopolysaccharide (LPS; Sigma-Aldrich) in the presence or absence of IL-4 (8 ng/ml; PeproTech) or IL-5 (10 ng/ml; PeproTech) for 48 h, and [^3^H]-thymidine (37 kBq) was added during the final 18 h of the culture. To assess PC differentiation *in vitro*, LN B cells (1 × 10^5^/well) were stimulated in complete RPMI medium with anti-IgM F(ab′)_2_ antibody (33 µg/ml; Jackson ImmunoResearch Laboratories), anti-CD40 antibody (5 µg/ml; BD Biosciences) or LPS (10 µg/ml; Sigma-Aldrich) in the presence or absence of IL-4 and IL-5 (both from PeproTech; 10 ng/ml) for 96 h, as described previously (32). In some experiments, CPYPP (33), a small-molecule inhibitor of DOCK2, was added to the culture at 12.5 µM.

### Flow Cytometry

The following antibodies and reagents were used at the indicated concentrations. Allophycocyanin (APC)-conjugated or phycoerythrin (PE)-conjugated anti-mouse CD45R/B220 (RA3-6B2; 2 µg/ml), fluorescein isothiocyanate (FITC)- or PE-conjugated anti-IgM (R6-60.2; 5 µg/ml), APC-anti-CD19 (1D3; 2 µg/ml), FITC-anti-IgD (11-26c.2a; 5 µg/ml), FITC-anti-CD21/CD35 (7G6; 10 µg/ml), biotinylated anti-heat stable antigen (HSA; M1/69; 0.1 µg/ml), FITC-anti-CD45.1 (A20; 2 µg/ml), PE-anti-CD38 (90; 0.7 µg/ml), PE-anti-IgG1 (A85-1; 1 µg/ml), PE-anti-CD138 (281-1; 2 µg/ml), PE-conjugated streptavidin (1 µg/ml) or PerCP-5.5cyanine-conjugated streptavidin (0.5 µg/ml) were from BD Bioscience. Biotinylated anti-GL7 (GL7; 2 µg/ml) purchased from eBioscience. Alexa Fluor 647-labeled HEL was prepared with an Alexa Fluor647 antibody-labeling kit (Invitrogen). Before staining with the antibodies, cells were incubated for 10 min on ice with anti-Fcγ III/II receptor (2.4G2; 0.5 µg/ml; BD Bioscience) to block Fc receptors. In some experiments, mice were injected intraperitoneally with 300 µl of BrdU (Invitrogen) and sacrificed 5 h later. Splenocytes were then stained with APC BrdU flow kit (Becton-Dickinson). Flow cytometric analyses were done on FACS Calibur (BD Bioscience).

### ELISA

Ninety six-well polystyrene plates (Thermo 3855) were coated overnight at 4°C with OVA (0.5 µg), HEL (2 µg), or goat anti-mouse Ig (IgM + IgG + IgA, H + L; #1010-01) antibody (Southern Biotech). After the wells were blocked with 150 µl phosphate-buffered saline (PBS) containing 1% sodium casein and 0.1% Tween-20, serial dilutions of sera were added. Alkaline phosphatase-conjugated isotype-specific antibodies (IgM, IgG1, IgG2b; Southern Biotech) were used to detect bound antibody. The reactions were visualized with the substrate p-nitrophenyl phosphate (Sigma-Aldrich) and detected at 405 nm.

### Plasmids and Transfection

The cDNA encoding HEL was amplified by PCR using the pET-22b HEL (amino acid residues 19–147) as a template (34). The following primers were used: 5′-ATGAGGTCTTTGCTAATCTTGGTGCTTTGCTTCCTGCCCCTGGCTGCTCTGGGGAAAGTCTTTGGACGATGTGAG-3′ and 5′-TCACAGCCGGCAGCCTCTGA-3′. The cDNAs encoding enhanced green fluorescent protein (EGFP) and the GPI anchor domain were prepared as described previously (35). The HEL-, EGFP-, and GPI anchor-coding cDNAs were cloned into the *EcoR* I-*Pst* I, *Pst* I-*BamH* I, and *BamH* I-*Not* I sites of the pBSSK vector, respectively, which was then cloned into the *Xho* I-*Not* I site of the pBJ1 vector. The pBJ1-HEL-GFP-GPI construct was linearized with *Sal* I and electroporated into the baby hamster kidney (BHK) cells expressing ICAM-1-GPI (36), together with pTRE2-puro vector. Cells were cultured in the presence of puromycin (0.3 µg/ml) and clones stably expressing HEL-GFP-GPI were selected.

### Pull-Down Assays and Immunoblotting

For Rac activation assays, LN B cells (1.25 × 10^7^ per sample) were stimulated with anti-IgM F(ab′)_2_ antibody (33 µg/ml; Jackson ImmunoResearch Laboratories) at 37°C for the specified times. Cells were then lysed by adding 1× MLB [Mg^2+^ Lysis Buffer: 25 mM Hepes (pH 7.5), 150 mM NaCl, 1% lgepal CA-630, 10 mM MgCl_2_, 1 mM EDTA, 10% glycerol; Millipore], followed by centrifugation at 20,000 × *g* for 1 min at 4°C. Aliquots were saved for total cell lysate controls, and the remaining lysates were incubated with agarose beads containing the GST-fusion Rac binding domain of PAK1 (#14-325; Millipore) at 4°C for 1 h. The beads were washed twice with 1× MLB buffer and suspended in 1× SDS-PAGE sample buffer [62.5 mM Tris–HCl (pH 6.8), 0.005% bromophenol blue, 2% SDS, 10% glycerol, 100 mM dithiothreitol]. The bound proteins and the same amount of total lysates were analyzed by SDS-PAGE, and blots were probed with the anti-Rac1 (23A8; Millipore) or Rac2 (3B10-2D9; Sigma-Aldrich) antibody.

To examine expression and activation of each signaling molecule, cells were lysed on ice in 20 mM Tris–HCl buffer (pH 7.5) containing 1% Triton X-100, 150 mM NaCl, 1 mM β-glycerophosphate, 1 mM Na_3_VO_4_, and complete™ protease inhibitors (Roche). After centrifugation, the supernatants were mixed with an equal volume of 2× SDS-PAGE sample buffer (125 mM Tris–HCl, 0.01% bromophenol blue, 4% SDS, 20% glycerol, and 200 mM dithiothreitol). Samples were boiled for 5 min and analyzed by immunoblotting with the following antibodies: rabbit anti-DOCK2 (09-454; 1:1,000 dilution; Millipore); rabbit anti-Syk (1:1,000 dilution; Cell Signaling Technology), rabbit anti-phospho-Syk (Tyr323; 1:1,000 dilution; Cell Signaling Technology), rabbit anti-p44/42 MAPK (Erk1/2) (1:1,000 dilution; Cell Signaling Technology), rabbit anti- phospho-p44/42 MAPK (Erk1/2) (Thr202/Tyr204; 1:1,000 dilution; Cell Signaling Technology), rabbit anti-Akt (1:1,000 dilution; Cell Signaling Technology), rabbit anti- phospho-Akt (Thr308; 1:1,000 dilution; Cell Signaling Technology), rabbit anti-BLNK (1:1,000 dilution; Cell Signaling Technology), rabbit anti- phospho-BLNK (Tyr96; 1:1,000 dilution; Cell Signaling Technology), rabbit anti-CD19 (1:1,000 dilution; Cell Signaling Technology), rabbit anti- phospho-CD19 (Tyr513; 1:1,000 dilution; Cell Signaling Technology). To analyze tyrosine phosphorylation of Vav or PLCγ2, cell extracts were incubated with protein G sepharose conjugated with anti-Vav (C-14; 1:1,000 dilution; Santa Cruz Biotechnology) or anti-PLCγ2 (Q-20; 1:1,000 dilution; Santa Cruz Biotechnology) antibody. The precipitates were subjected to immunoblotting using anti-phosphotyrosine antibody (pY99; 1:1,000 dilution; Santa Cruz Biotechnology).

### Calcium Flux Assays

Lymph node B cells (1 × 10^6^) were loaded with 3 µM Fura 2-AM (Wako Chemicals) for 30 min at 37°C. Cells were then resuspended in Hank’s buffered salt solution containing calcium and magnesium, and were stimulated with anti-IgM F(ab′)_2_ antibody (33 µg/ml). Fluorescence intensities were monitored at an excitation wavelength of 340 or 380 nm and emission wavelength of 510 nm using a Flex Station3 (Molecular Devices). Ionomycin (10 µM; Sigma-Aldrich) was used as a positive control.

### Assays for IS Formation

To analyze IS formation, LN B cells (3 × 10^5^) from HyHEL10 mice were stained with PKH26 (Sigma-Aldrich) or biotinylated anti-LFA-1 antibody (2D7; BD Biosciences) followed by Alexa546-conjugated streptavidin (Invitrogen) before assays. After LN B cells were incubated on a monolayer of BHK cells expressing ICAM1-GPI and HEL-GFP-GPI (designated BHK-ICAM-HEL cells) at 37°C for the specified times, cells were fixed with 4% paraformaldehyde for 12 min. All images were taken with FV3000 laser scanning confocal microscopy (Olympus).

### Adoptive Transfers and Immunization

HEL was covalently conjugated to sheep red blood cell (SRBC; KOHJIN BIO) with 1-ethyl-3-(3-dimethylaminopropyl) carbodiimide hydrochloride (Sigma-Aldrich) as described previously (37). CD45.1^+^ LN B cells (1 × 10^5^ B cells from *Dock2^+/−^* HyHEL10 mice or 2 × 10^5^ B cells from *Dock2^−/−^* HyHEL10 mice) were adoptively transferred into 6- to 7-week-old C57BL/6 mice (CD45.2^+^) together with 2 × 10^8^ HEL-SRBC or SRBC alone. Mice were sacrificed at the specified time points, and spleen cells were analyzed by flow cytometry. In some experiments, mice were injected intraperitoneally with 300 µl of BrdU (Invitrogen) to assess cell proliferation.

To examine antigen-specific antibody responses, mice were immunized by intraperitoneal injection of ovalbumin (OVA; 50 µg per mouse; Sigma-Aldrich) emulsified in complete Freund’s adjuvant (CFA; Difco Laboratories). Fourteen days later, the serum levels of anti-OVA antibody were determined by ELISA.

### Immunohistochemical Analyses

Freshly prepared spleens were embedded in Tissue-Tek OCT compound (Sakura Finetechnical), and frozen at −80°C. Cryosections (10 µm) were fixed with 4% (w/v) paraformaldehyde for 10 min at 37°C. After being blocked with 10% horse serum (Sigma-Aldrich), samples were stained with FITC-conjugated anti-B220 (RA3-6B2; BD Biosciences), PE-conjugated anti-CD3 (17A2; BioLegend), anti-metallophillic macrophages (MOMA1; BMA Biomedicals) followed by Alexa 647-conjugated goat anti-rat antibody (Invitrogen). All images were obtained with a laser-scanning confocal microscope (LSM510 META, Carl Zeiss).

### Reverse Transcription (RT)-PCR

Total RNA was isolated using ISOGEN (Nippon Gene). After treatment with RNase-free DNase I (Life Technologies), RNA samples were reverse-transcribed with oligo(dT) primers (Life Technologies) and SuperScript III reverse transcriptase (Life Technologies) for amplification by PCR. The following PCR primers were used: *Prdm1*; 5′-GACTGGGTGGACATGAGAGAG-3′ and 5′-CCATCAATGAAGTGGTGGAAC-3′. *Gapdh*; 5′-ACCACAGTCCATGCCATCAC-3′ and 5′-TCCACCACCCTGTTGCTGTA-3′.

### Homing Assays

B cells were purified from the LNs from *Dock2^+/+^* and *Dock2^−/−^* mice and labeled with PKH26 fluorescent cell linkers (Sigma-Aldrich) or CMTMR (Life Technologies), respectively. After intravenous injection of LN B cells (1–2 × 10^7^) into C57BL/6 mice, the ratio of transferred B cells in the white pulp was compared at 48 h later.

### Statistical Analyses

Statistical analyses were performed using GraphPad Prism. The data was initially tested with a Kolmogorov–Smirnov test for normal distribution. Parametric data were analyzed using a two-tailed unpaired Student’s *t-*test when two groups were compared. Nonparametric data were analyzed with a two-tailed Mann–Whitney test when two groups were compared. *P*-values less than 0.05 were considered significant.

## Results

### DOCK2 Is a Major Rac GEF Acting Downstream of BCR

**Figure 1:**
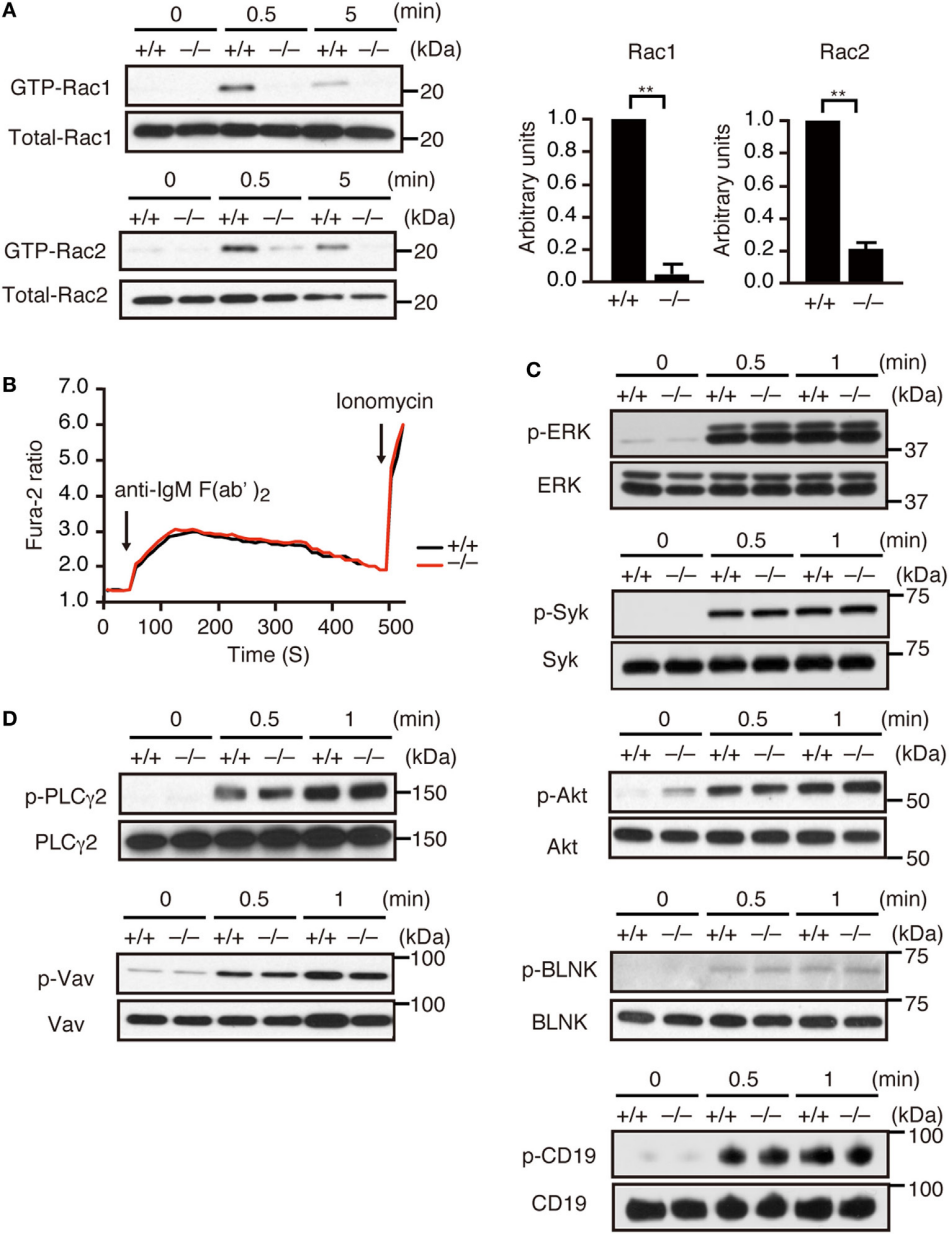
DOCK2 is a major Rac GEF acting downstream of B cell receptor (BCR). **(A)** BCR-mediated activation of Rac1 and Rac2 were compared between *Dock2^+/+^* and *Dock2^−/−^* lymph node (LN) B cells. Results were quantified by densitometry and are expressed as the ratio of GTP-bound form to the total protein after normalization of the 0.5 min-value of *Dock2^+/+^* samples to an arbitrary value of 1. Data for 0.5 min are indicated as the mean ± SD of five independent experiments. **p < 0.01 (two-tailed Mann–Whitney test). **(B)** Fura-2-loaded *Dock2^+/+^* and *Dock2^−/−^* LN B cells were stimulated with anti-IgM F(ab′)_2_ antibody or ionomycin. Data are indicated as the Fura-2 ratio at 340:380 nm and are representative of three independent experiments. **(C,D)** *Dock2^+/+^* and *Dock2^−/−^* LN B cells were stimulated with anti-IgM F(ab′)_2_ antibody and analyzed for phosphorylation of each molecule. In **(C)**, phosphorylations of ERK, Syk, Akt, BLNK, and CD19 were analyzed using phosphorylation-specific antibodies. In **(D)**, cell extracts were immunoprecipitated with anti-Vav or anti-PLCγ2 antibody and analyzed with anti-phosphotyrosine antibody.

Although *Dock2^−/−^* mice exhibited diminished numbers of transitional B cells and mature follicular B cells in the spleen (Figures S2B,C in Supplementary Material), LN B cells from C57BL/6 (designated *Dock2^+/+^*) mice and *Dock2^−/−^* mice showed similar IgM^low^IgD^hi^ mature phenotype due to the lack of transitional B cells (2) (Figure S2D in Supplementary Material). Therefore, to examine whether DOCK2 functions downstream of BCR, we prepared LN B cells and analyzed activation and phosphorylation of the signaling molecules. When LN B cells from *Dock2^+/+^* mice were stimulated with anti-IgM F(ab′)_2_ antibody, the GTP-bound, activated Rac1 and Rac2 were readily detected at 0.5 min after stimulation (Figure 1A). However, BCR-mediated activation of Rac1 and Rac2 were reduced in *Dock2^−/−^* B cells to 4.7 and 20.9% of the wild-type (WT) levels, respectively (Figure 1A). These results indicate that DOCK2 is a major Rac GEF acting downstream of BCR. On the other hand, BCR-mediated calcium influx occurred normally even in *Dock2^−/−^* B cells (Figure 1B). In addition, we found that DOCK2 deficiency did not affect phosphorylations of other signaling molecules such as Erk, Syk, Akt, BLNK, CD19, PLCγ2, and Vav (Figures 1C,D).

### DOCK2 Regulates BCR-Mediated B Cell Proliferation and PC Differentiation *In Vitro*

**Figure 2:**
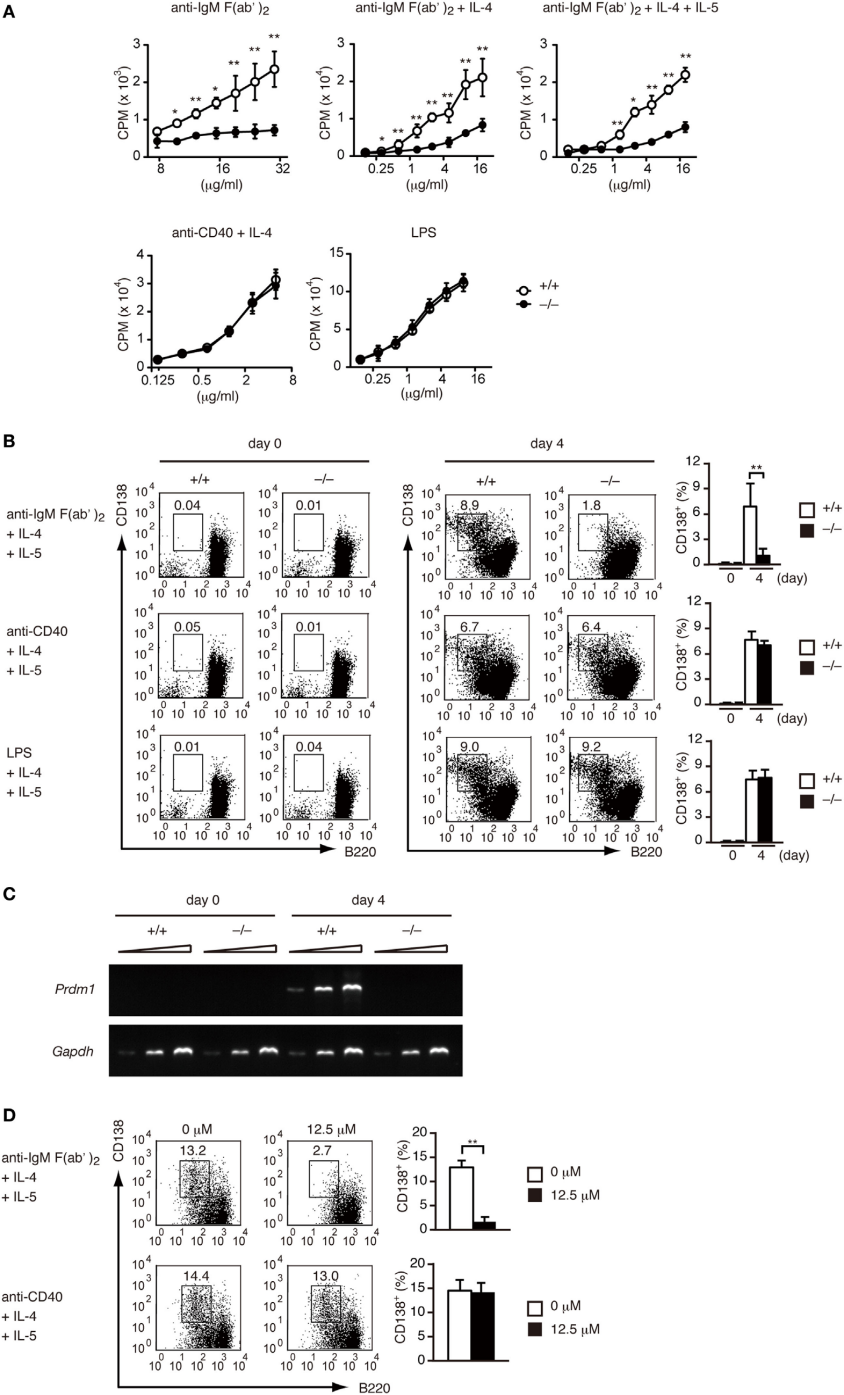
DOCK2 regulates B cell receptor-mediated B cell proliferation and PC differentiation *in vitro*. **(A)** *Dock2^+/+^* and *Dock2^−/−^* LN B cells were stimulated with anti-IgM F(ab′)_2_ antibody, anti-CD40 antibody, or lipopolysaccharide at the indicated concentrations in the presence or absence of IL-4/IL-5, and B cell proliferation was analyzed. Data are indicated as the mean ± SD of five independent experiments. *p < 0.05; **p < 0.01 (two-tailed Mann–Whitney test). **(B)** Following stimulation with anti-IgM F(ab′)_2_ or anti-CD40 antibody in the presence of IL-4 and IL-5 for 4 days, *Dock2^+/+^* and *Dock2^−/−^* LN B cells were analyzed for the expression of CD138 and B220 to assess PC differentiation. FACS profiles at day 0 and day 4 after stimulation are shown. Data are indicated as the mean ± SD of five independent experiments. **p < 0.01 (two-tailed unpaired Student’s *t*-test). **(C)** Following stimulation of *Dock2^+/+^* and *Dock2^−/−^* LN B cells with anti-IgM F(ab′)_2_ antibody in the presence of IL-4 and IL-5, the expression of *Prdm1* and *Gapdh* were analyzed with reverse transcription-PCR. Amplification increased by three cycles from the left to the right starting at 28 cycles for *Prdm1* or 20 cycles for *Gapdh*. Data are representative of three independent experiments. **(D)** The effect of CPYPP (12.5 µM) on *in vitro* PC differentiation was analyzed as in **(B)**. Data are indicated as the mean ± SD of seven independent experiments. **p < 0.01 (two-tailed Mann–Whitney test).

Having found that DOCK2 acts downstream of BCR, we next examined whether DOCK2 deficiency affects BCR-mediated B cell functions *in vitro*. Although *Dock2^+/+^* B cells proliferated vigorously when stimulated with anti-IgM F(ab′)_2_ antibody in the presence or absence of IL-4/IL-5, BCR-mediated B-cell proliferation was impaired in the absence of DOCK2 (Figure 2A). When *Dock2^+/+^* B cells were stimulated with anti-IgM F(ab′)_2_ antibody plus IL-4 and IL-5 for 4 days, they efficiently differentiated into CD138^+^ PCs (Figure 2B). However, in the case of *Dock2^−/−^* B cells, CD138^+^ PCs were hardly detected under the same culture condition (Figure 2B). Consistent with this, the expression of *Prdm1*, which encodes the transcription factor Blimp-1 important for PC differentiation (38), was readily detected in *Dock2^+/+^* B cells, but not *Dock2^−/−^* B cells (Figure 2C). Importantly, BCR-mediated PC differentiation was impaired when *Dock2^+/+^* B cells were treated with CPYPP (Figure 2D), a small-molecule inhibitor of DOCK2 that binds to the DOCK2 DHR-2 domain and inhibits its Rac GEF activity (33). On the other hand, DOCK2 deficiency did not affect B-cell proliferation and PC differentiation in response to CD40 ligation or LPS stimulation (Figures 2A,B). Thus, DOCK2 selectively regulates BCR-mediated B cell proliferation and PC differentiation *via* Rac activation.

### DOCK2 Regulates BCR-Mediated IS Formation

**Figure 3:**
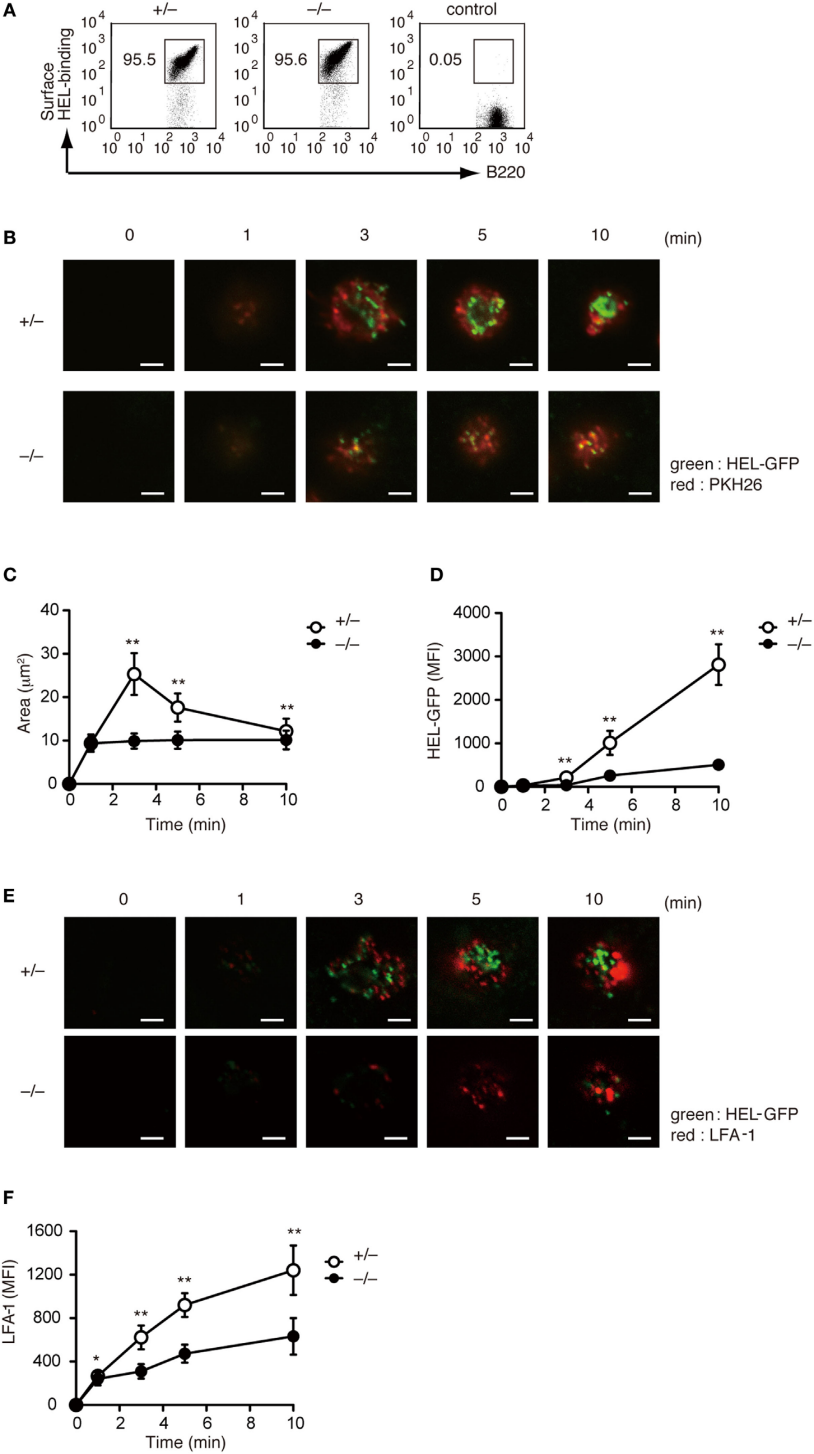
DOCK2 regulates immunological synapse formation. **(A)** FACS profiles showing comparable binding of HEL to *Dock2^+/−^* and *Dock2^−/−^* LN B cells. LN B cells from C57BL/6 mice were used as control samples. **(B–D)** Following incubation of *Dock2^+/−^* and *Dock2^−/−^* HyHEL10 mice with baby hamster kidney (BHK)-ICAM-HEL, the area of B cell contact **(C)** and the mean fluorescence intensity (MFI) of HEL-GFP **(D)** were compared at the indicated time points. Data are indicated as the mean ± SD of 60 cells collected from three separate experiments. **p < 0.01 (two-tailed Mann–Whitney test). **(E,F)** Following incubation of *Dock2^+/−^* and *Dock2^−/−^* HyHEL10 mice with BHK-ICAM-HEL, the MFI of leukocyte function-associated antigen-1 (LFA-1) **(F)** was compared at the indicated time points. Data are indicated as the mean ± SD of 60 cells collected from three separate experiments. *p < 0.05; **p < 0.01 (two-tailed Mann–Whitney test).

Although a previous study has indicated that B cell adhesion and IS formation are impaired in Rac2-deficient B cells (18), the physiological function of Rac1 and Rac2 activation in this process is not completely understood. To address this issue, we crossed *Dock2^−/−^* mice with HyHEL10 mice that express a defined anti-HEL BCR and are capable of normal Ig class-switch recombination and somatic hypermutation. Irrespective of DOCK2 expression, LN B cells from HyHEL10 mice comparably bound to HEL (Figure 3A). When PKH26-labeled LN B cells from *Dock2^+/−^* HyHEL10 mice were incubated with BHK-ICAM-HEL cells, they rapidly spread over the target membrane, where small clusters of GFP-fusion HEL were formed by 3 min within the area of interaction (Figure 3B). However, in the case of *Dock2^−/−^* HyHEL10 B cells, a spreading response was impaired with a significant reduction of the number of BCR microclusters at the site of the contact (Figures 3B–D). Similarly, LFA-1 accumulation was reduced in the case of *Dock2^−/−^* HyHEL10 B cells (Figures 3E,F). These results indicate that BCR-mediated IS formation critically depends on DOCK2.

### DOCK2 Is Required for Expansion of GC B Cells and Differentiation into PCs in Adoptive Transfer Model

**Figure 4:**
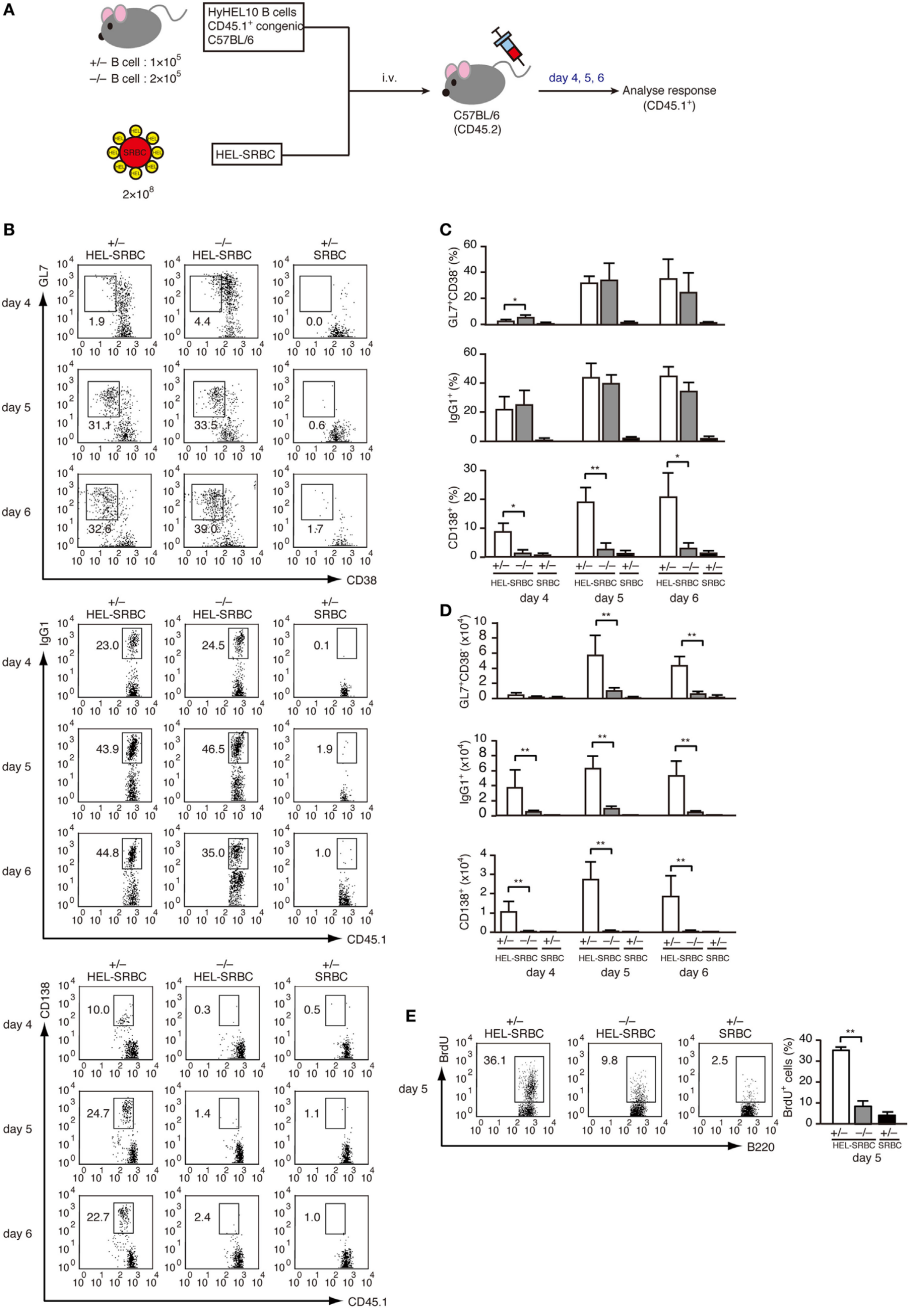
DOCK2 is required for germinal center (GC) B cell expansion and PC differentiation in adoptive transfer model. **(A)** Schematic representation of the adoptive transfer model used in this study. **(B)** FACS profiles indicating the expression of GL7, CD38, IgG1, and/or CD138 in CD45.1^+^ transferred B cells from *Dock2^+/−^* and *Dock2^−/−^* HyHEL10 mice. Data were obtained at day 4, day 5, and day 6 after transfer and are representative of five independent experiments. **(C)** Following adoptive transfer, the percentages of GL7^+^CD38^−^ GC B cells, IgG1^+^ B cells, CD138^+^ plasma cells (PCs) in CD45.1^+^ transferred B cells were compared at the indicated time points between *Dock2^+/−^* and *Dock2^−/−^* HyHEL10 mice. Data are indicated as the mean ± SD of five independent experiments. *p < 0.05; **p < 0.01 (two-tailed Mann–Whitney test). **(D)** Following adoptive transfer, the numbers of GL7^+^CD38^−^ GC B cells, IgG1^+^ B cells, CD138^+^ PCs were compared at the indicated time points between *Dock2^+/−^* and *Dock2^−/−^* HyHEL10 mice. Data are indicated as the mean ± SD of five independent experiments. **p < 0.01 (two-tailed Mann–Whitney test). **(E)** The percentages of BrdU^+^ B cells in CD45.1^+^ transferred B cells were compared between *Dock2^+/−^* and *Dock2^−/−^* HyHEL10 mice 5 days after adoptive transfer. Data are indicated as the mean ± SD of nine independent experiments. **p < 0.01 (two-tailed Mann–Whitney test).

To examine the role of DOCK2 in PC differentiation *in vivo*, we prepared LN CD45.1^+^ B cells from *Dock2^+/−^* and *Dock2^−/−^* HyHEL10 mice and adoptively transferred them into C57BL/6 mice (CD45.2) with HEL-conjugated SRBCs (Figure 4A). As DOCK2 deficiency reduces B cell homing to the secondary lymphoid organs (26, 39), we injected *Dock2^−/−^* B cells twice as much as *Dock2^+/−^* B cells to compensate the number of B cells in the lymphoid follicle (Figure S3 in Supplementary Material). In both cases, the frequencies of GL7^+^CD38^−^ B cells and IgG1^+^ B cells to the total CD45.1^+^ B cells were comparable between *Dock2^+/−^* and *Dock2^−/−^* B cells at day 5 and day 6 after transfer (Figures 4B,C), indicating that DOCK2 deficiency does not affect differentiation of antigen-engaged B cells to GC B cells and class-switch recombination. However, while *Dock2^+/−^* GC B cells proliferated well from day 4 to day 5, such expansion was impaired in the case of *Dock2^−/−^* GC B cells (Figure 4D). This was further supported by analyzing BrdU incorporation (Figure 4E). More importantly, we found that B cells from *Dock2^−/−^* HyHEL10 mice failed to differentiate efficiently to CD138^+^ PCs (Figures 4B–D). These results indicate that DOCK2 is required for expansion of GC B cells and differentiation into PCs during TD antibody response.

### Development and Characterization of Conditional KO Mice Lacking DOCK2 in a B Cell-Specific Manner

**Figure 5:**
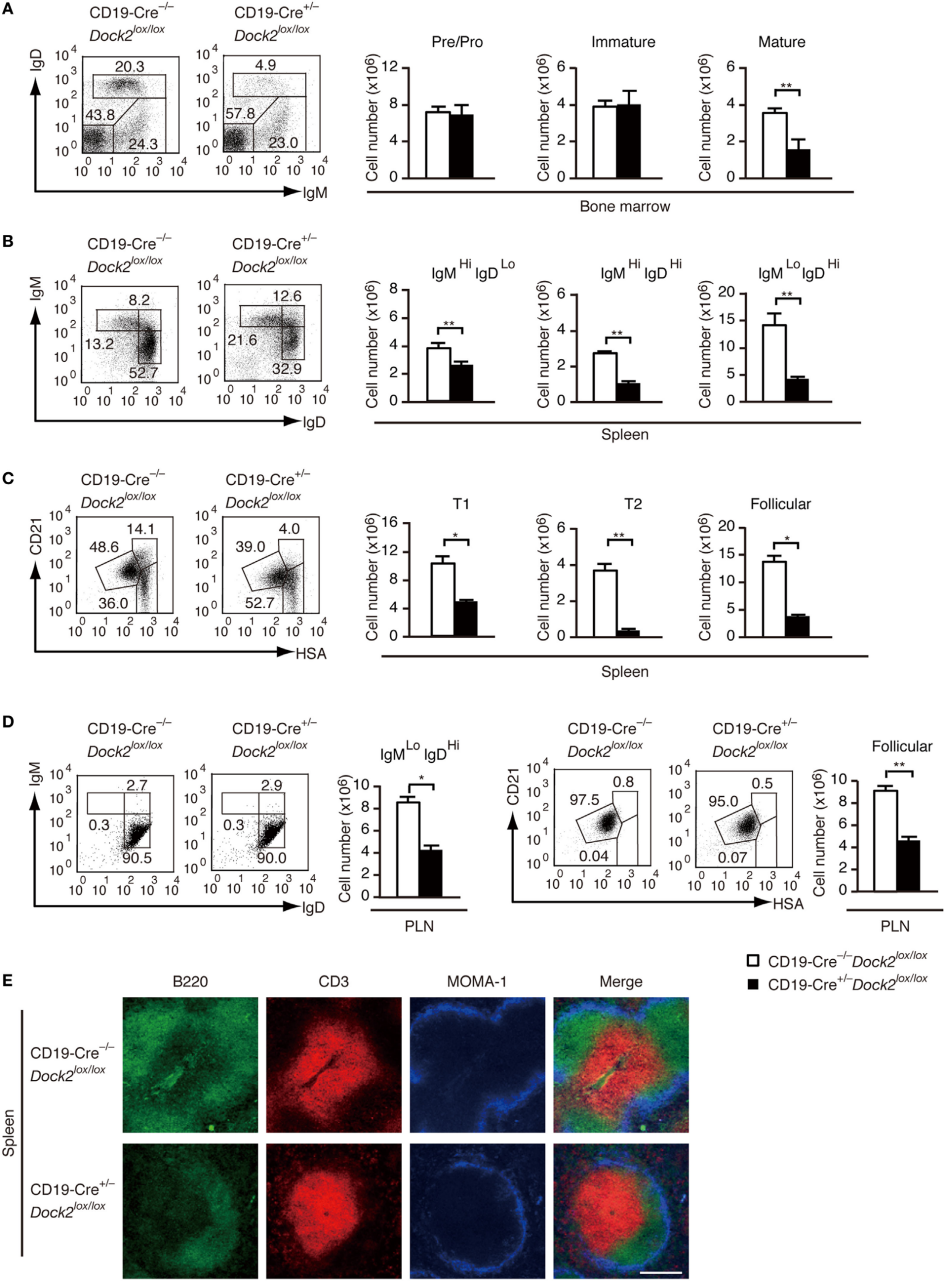
Characterization of CD19-Cre*^+/−^ Dock2^lox/lox^* mice. **(A)** FACS profiles for expression of IgM and IgD in the CD19^+^ bone marrow B cells. The number of each subset of B cells was compared between CD19-Cre*^−/−^ Dock2^lox/lox^* and CD19-Cre*^+/−^ Dock2^lox/lox^* mice. Data are indicated as the mean ± SD of five mice. **p < 0.01 (two-tailed Mann–Whitney test). **(B)** FACS profiles for expression of IgM and IgD in the B220^+^ splenic B cells. The number of each subset of B cells was compared between CD19-Cre*^−/−^ Dock2^lox/lox^* and CD19-Cre*^+/−^ Dock2^lox/lox^* mice. Data are indicated as the mean ± SD of 5 mice. **p < 0.01 (two-tailed unpaired Student’s *t*-test). **(C)** FACS profiles for expression of CD21 and heat stable antigen (HSA) in the B220^+^ splenic B cells. The number of each subset of B cells (T1, T2, and follicular B cells) was compared between CD19-Cre^–/–^ *Dock2^lox/lox^* and CD19-Cre*^+/−^ Dock2^lox/lox^* mice. Data are indicated as the mean ± SD of five mice. *p < 0.05; **p < 0.01 (two-tailed Mann–Whitney test). **(D)** FACS profiles for expression of IgM and IgD or CD21 and HSA in the B220^+^ peripheral LN (PLN) B cells. The number of each subset of B cells was compared between CD19-Cre*^−/−^ Dock2^lox/lox^* and CD19-Cre*^+/−^ Dock2^lox/lox^* mice. Data are indicated as the mean ± SD of five mice. *p < 0.05; **p < 0.01 (two-tailed Mann–Whitney test). **(E)** Immunohistochemical analyses of the spleen sections from CD19-Cre*^−/−^ Dock2^lox/lox^* and CD19-Cre*^+/−^ Dock2^lox/lox^* mice.

To examine the B cell intrinsic role of DOCK2 under more physiological condition, we developed conditional KO mice lacking DOCK2 in a B cell-specific manner (CD19-Cre*^+/−^Dock2^lox/lox^* mice). Western blot analyses revealed that DOCK2 expression was selectively deleted in B-lineage cells in these mice (Figure S4 in Supplementary Material). We first compared B cell development between CD19-Cre*^+/−^Dock2^lox/lox^* and CD19-Cre*^−/−^Dock2^lox/lox^* mice. Although the amounts of pre/pro B cells and immature B cells in the BM were unchanged between them (Figure 5A), the number of mature recirculating B cells was reduced to 44% of the control level (Figure 5A). Similarly, CD19-Cre*^+/−^Dock2^lox/lox^* mice had diminished numbers of transitional B cells and mature follicular B cells in the spleen (Figures 5B,C), as seen in *Dock2^−/−^* mice (Figures S2B,C in Supplementary Material). On the other hand, no phenotypic difference was found when LN B cells from CD19-Cre*^+/−^Dock2^lox/lox^* and CD19-Cre*^−/−^Dock2^lox/lox^* mice were stained for IgM and IgD, or CD21 and HSA (Figure 5D). These phenotypes were similar to those of *Dock2^−/−^* mice (Figure S2D in Supplementary Material). Consistent with the FACS data, immunohistochemical analyses of the spleen revealed that the relative size and number of B cell follicles was significantly reduced in CD19-Cre*^+/−^Dock2^lox/lox^* mice, compared with CD19-Cre^–/–^ *Dock2^lox/lox^* mice (Figure 5E). However, the organization of T cells and macrophages in the white pulp was not altered in CD19-Cre*^+/−^Dock2^lox/lox^* mice (Figure 5E).

### A Critical Role of DOCK2 in IgG Antibody Responses *In Vivo*

**Figure 6:**
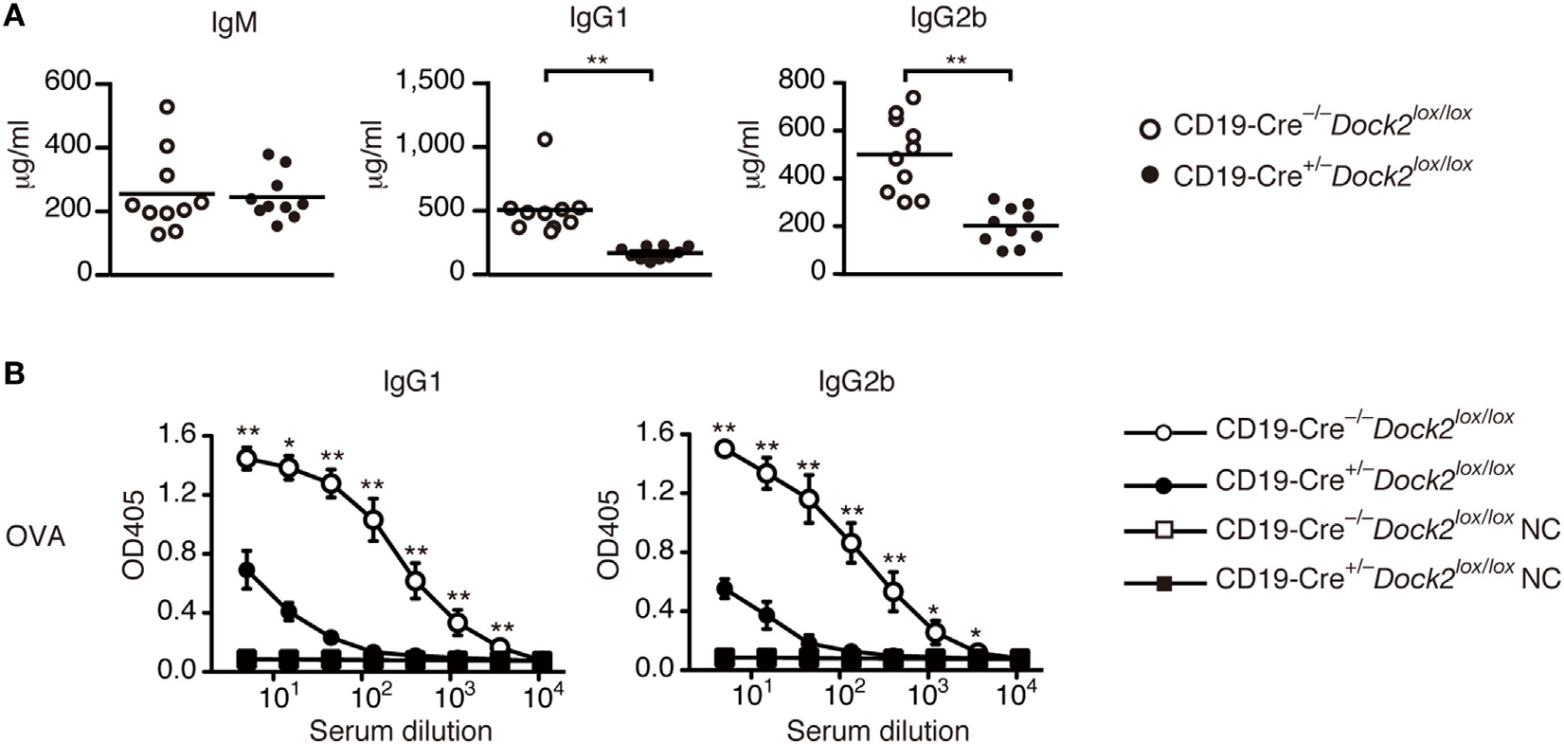
Defective antibody production in CD19-Cre*^+/−^ Dock2^lox/lox^* mice. **(A)** Comparison of serum IgM, IgG1 and IgG2b in CD19-Cre*^−/−^ Dock2^lox/lox^* and CD19-Cre*^+/−^ Dock2^lox/lox^* mice under the steady state. Data are indicated as the mean ± SD of 10 mice. **p < 0.01 (two-tailed Mann–Whitney test). **(B)** OVA-specific antibody production was compared between CD19-Cre*^−/−^ Dock2^lox/lox^* and CD19-Cre*^+/−^ Dock2^lox/lox^* mice at day 14 after immunization. For negative controls, wells were coated with HEL. Data are indicated as the mean ± SD of five independent experiments. *p < 0.05; **p < 0.01 (two-tailed Mann–Whitney test).

Under basal condition, serum levels of IgG1 and IgG2b were significantly reduced in CD19-Cre*^+/−^Dock2^lox/lox^* mice, compared with CD19-Cre*^−/−^Dock2^lox/lox^* control mice (Figure 6A). We then compared TD antibody response between CD19-Cre*^+/−^Dock2^lox/lox^* and CD19-Cre*^−/−^Dock2^lox/lox^* mice. When CD19-Cre*^−/−^Dock2^lox/lox^* mice were injected intraperitoneally with OVA in CFA, antigen-specific IgG1 and IgG2b antibodies were readily detected at 14 days after immunization (Figure 6B). However, OVA-specific IgG antibody production was severely impaired in CD19-Cre*^+/−^Dock2^lox/lox^* mice (Figure 6B). These results demonstrate a critical role of DOCK2 in IgG antibody responses *in vivo*.

## Discussion

DOCK2 regulates B cell migration and adhesion by acting downstream of chemokine receptors (26, 39), yet, its role in BCR signaling is poorly understood. Here, we have shown that activations of Rac1 and Rac2 following BCR stimulation were markedly reduced in the absence of DOCK2. Our results thus identify DOCK2 as a key Rac GEF acting downstream of BCR. So far, the DH-type GEFs Vav proteins (Vav1, Vav2, and Vav3) have been considered to regulate B cell functions as the Rac GEFs (40, 41). Although tyrosine phosphorylation of Vav augments its Rac GEF activity (42), BCR-mediated Vav phosphorylation was unchanged between WT and *Dock2^−/−^* B cells. In addition, DOCK2 deficiency did not affect BCR-mediated calcium influx, which is defective in B cells from *Vav1^−/−^Vav2^−/−^* double KO mice (40, 41). The precise relationship between DOCK2 and Vav proteins in BCR signaling is currently unknown. However, recent studies have shown that Vav proteins play important roles in T cells and NK cells independently of the Rac GEF activities (43, 44). In light of this, it seems likely that Vav proteins act as adaptor molecules and regulate B cell functions *via* calcium mobilization.

Although Rac activation has been implicated in BCR-mediated IS formation (4, 18), its physiological relevance and the upstream signaling cascade are not completely understood. We found that antigen-driven B cell spreading and sustained growth of BCR microclusters were impaired in *Dock2^−/−^* primary B cells. As these cellular responses are abrogated by actin polymerization inhibitors (4, 45), Rac activation-induced remodeling of the actin cytoskeleton is likely to be involved. In addition, a recent study using chicken DT40 B cells revealed that the growth of BCR microclusters critically depends on PIP_3_, a lipid product of phosphatidylinositol 3-kinases (PI3Ks) (46). Indeed, DOCK2 binds to PIP_3_ through its DHR-1 domain (21, 23). Therefore, it is highly conceivable that PI3K activity is required to recruit DOCK2 to the synaptic membrane and activate Rac locally for IS formation, as seen in other lymphocytes (35, 46, 47). While DOCK2 deficiency leads to defective IS formation, it did not affect phosphorylation of major signaling molecules downstream of BCR stimulated with a soluble cross-linking antibody. The role of DOCK2 in signal transduction might be more critical in the *in vivo* situations where antigen concentrations are often low and the signaling induced by antigen presented on the membrane with adhesion molecules becomes more important.

In this study, we have also shown that in the absence of DOCK2, BCR-mediated PC differentiation was severely impaired *in vitro* and *in vivo*. Similar results were obtained when WT B cells were treated with CPYPP, which binds to the DOCK2 DHR-2 domain and inhibits its Rac GEF activity (33). These results indicate that DOCK2 regulates BCR-mediated PC differentiation through Rac activation. How DOCK2-Rac signaling axis regulates PC differentiation remains to be determined. However, accumulating evidence indicates that low affinity antigens fail to induce PC differentiation (8–10). As B cell spreading and growth of BCR microclusters act to increase the number of signalosomes within the membrane (4), their defects in *Dock2^−/−^* B cells may lead to the failure to amplify signaling above the threshold required for PC differentiation. Alternatively, in light of the fact that Rac has direct roles in the regulation of gene transcription (48, 49), activated Rac may be involved in the expression of *Prdm1* or its related genes during PC differentiation. Also, it may be possible that DOCK2–Rac axis regulates the expression of other molecules required for survival, growth, or differentiation during PC differentiation, because it has been reported that DOCK2 deficiency affects helper T cell differentiation by modulating cytokine receptor expression (50).

Finally, we have shown that CD19-Cre^+/−^*Dock2^lox/lox^* conditional KO mice fail to mount antigen-specific IgG antibody upon immunization of OVA. This result is in marked contrast to a recent study showing that after treatment with tamoxifen to delete Rac1 in Rac2^–/–^ B cells, Mb1-Cre-ERT2 *Rac1^lox/lox^Rac2^–/–^* mice exhibited increased IgG1 and IgG2b antibody to a TD antigen (51). As DOCK2 was deleted early during B cell development in CD19-Cre^+/−^*Dock2^lox/lox^* mice, B cell trafficking is also impaired in this model. On the other hand, there is a time lag between the last tamoxifen treatment and antibody measurement in Mb1-Cre-ERT2 *Rac1^lox/lox^Rac2^−/−^* mice. These differences may affect the outcome of antibody production to TD antigens. Alternatively, it would be possible to speculate that genetic loss of Rac and functional loss of activated Rac are not essentially the same in terms of the regulation of humoral immunity. Further analyses are needed to determine the underlying mechanisms.

## Ethics Statement

Mice were maintained under specific-pathogen-free conditions in the animal facility of Kyushu University. The protocol of animal experiments was performed in accordance with the guidelines of the committee of Ethics on Animal Experiments of Kyushu University.

## Author Contributions

MU, TU, AN, and FS performed functional, histological, and biochemical analyses; YK and DS performed *in vivo* experiments; KK contributed to histological analyses; TO provided reagents; MU, TU, and TO contributed to writing the manuscript; TU and YF conceived the project, interpreted the data, and wrote the manuscript.

## Conflict of Interest Statement

The authors declare that the research was conducted in the absence of any commercial or financial relationships that could be construed as a potential conflict of interest.
