# Supplementary material for: The Rac Activator DOCK2 Mediates Plasma Cell Differentiation and IgG Antibody Production
Source: Front Immunol. 2018 Feb 16;9:243. doi: 10.3389/fimmu.2018.00243 (PMC5820292; doi:10.3389/fimmu.2018.00243)
Supplement: Supplementary file 1 [file Presentation_1.PDF]

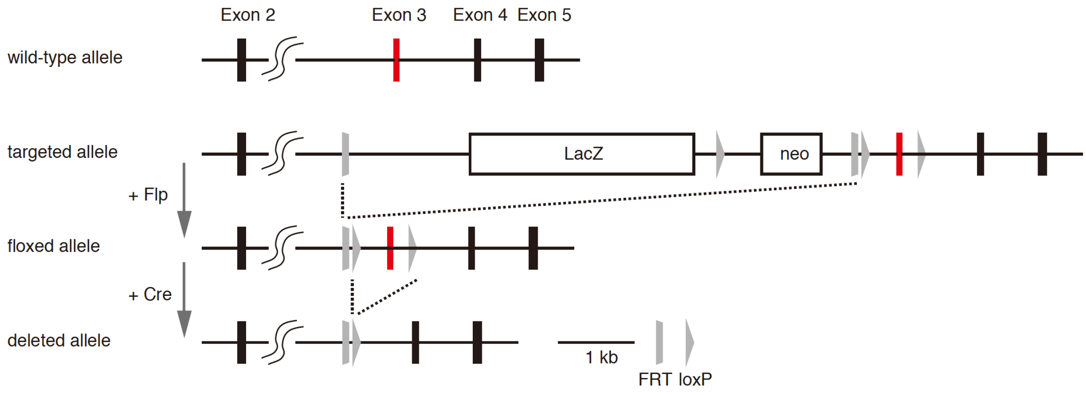

FIGURE S1 | Strategy used for conditional deletion of *Dock2* allele. The flippase recognition target (FRT)-flanked neomycin resistance gene (Neo) was removed by breeding with CAG-FLPe transgenic mice expressing flippase (Flp) (1). Then, the loxP-flanked exon 3 was removed by Cre to inactivate the *Dock2* gene.

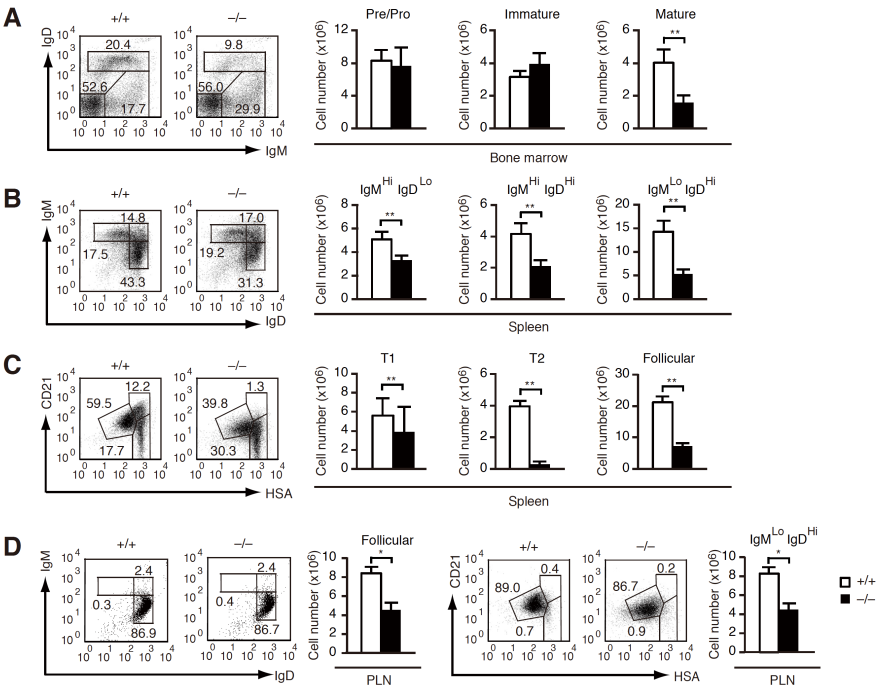

FIGURE S2 | Defective B cell development in *Dock2*$^{-/-}$ mice. **(A)** FACS profiles for expression of IgM and IgD in the CD19^{+} BM B cells. The number of each subset of B cells was compared between *Dock2*$^{+/+}$ and *Dock2*$^{-/-}$ mice. Data are indicated as the mean ± S.D. of 5 mice. **$p < 0.01$ (two-tailed unpaired Student's t test). **(B)** FACS profiles for expression of IgM and IgD in the B220^{+} splenic B cells. The number of each subset of B cells was compared between *Dock2*$^{+/+}$ and *Dock2*$^{-/-}$ mice. Data are indicated as the mean ± S.D. of 5 mice. **$p < 0.01$ (two-tailed unpaired Student's t test). **(C)** FACS profiles for expression of CD21 and HSA in the B220^{+} splenic B cells. The number of each subset of B cells (T1, T2 and follicular B cells) was compared between *Dock2*$^{+/+}$ and *Dock2*$^{-/-}$ mice. Data are indicated as the mean ± S.D. of 5 mice. **$p < 0.01$ (two-tailed unpaired Student's t test). **(D)** FACS profiles for expression of IgM and IgD or CD21 and HSA in the B220^{+} LN B cells. The number of each subset of B cells was compared between *Dock2*$^{+/+}$ and *Dock2*$^{-/-}$ mice. Data are indicated as the mean ± S.D. of 5 mice. *$p < 0.05$ (two-tailed Mann-Whitney test).

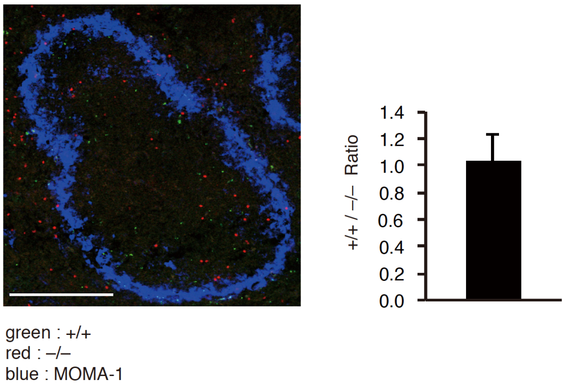

FIGURE S3 | Homing assays showing that comparable numbers of *Dock2*$^{+/+}$ and *Dock2*$^{-/-}$ B cells exist in the spleen after adoptive transfer. After intravenous injection of LN B cells (1×10^7 for *Dock2*$^{+/+}$ B cells and 2×10^7 for *Dock2*$^{-/-}$ B cells) into C57BL/6 mice, the ratio of transferred B cells in the white pulp was compared at 48 h later. Data are indicated as the mean ± S.D. of three independent experiments.

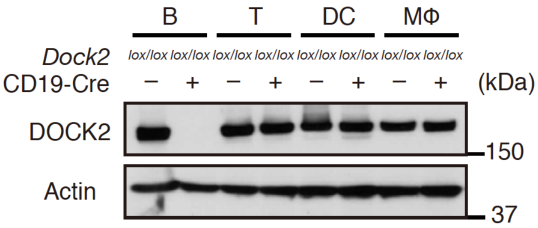

FIGURE S4 | Western blot analyses showing a B cell-specific deletion of DOCK2 in CD19-Cre$^{+/-}$ *Dock2*$^{lox/lox}$ mice. Cell extracts were prepared from B cells, T cells, dendritic cells (DC) and macrophages (Mϕ), and DOCK2 expression was analyzed by immunoblotting.

REFERENCE

1. Kanki H, Suzuki H, Itohara S. High-efficiency CAG-FLPe deleter mice in C57BL/6J background. *Exp Anim* (2006) **55**(2):137-41. http://doi.org/10.1538/expanim.55.137
